# Dog bite- fracture of the mandible in a 9 month old infant: a case report

**DOI:** 10.1186/1757-1626-2-44

**Published:** 2009-01-12

**Authors:** TWM Walker, PC Modayil, L Cascarini, JC Collyer

**Affiliations:** 1 Department of Oral & Maxillofacial Surgery, Queen Victoria Hospital, Holtye Road, East Grinstead, RH19 3DZ, UK

## Abstract

**Background:**

We present the case of a fractured mandible due to a dog bite in a 9 month old female. Dog bites in this age group are rare as are fractured mandibles. There are only two reported cases of fractured mandibles due to dog bites in the literature. This is the youngest. The other reported cases were in a 1 year old and also in a 4 year old.

**Case Presentation:**

A 9 month old female was brought by her parents to the Emergency Department after sustaining a dog bit to the face. This was assessed by the emergency physicians and deemed to be superficial. The patients wounds were irrigated, and she was given oral antibiotics. She was transferred to our department were she was assessed under anaesthetic. A fracture of her mandible was discovered and treated with open reduction and internal fixation.

**Conclusion:**

The case presentation highlights the important of proper assessment of facial lacerations for not only neurovascular status and the parotid duct, but also the hard tissues. The case also highlights the difficulty of treating children and infants with fractures of the mandible and the importance of follow-up to monitor growth.

## Background

Bite wounds are among the most common trauma to which man is subject to. In urban areas the main suspects are dogs, cats and humans [1]. Dog bites have a recognised mortality and there are at least 15 deaths per year in the United States [2]. In the young the most documented injuries have been to the face, head and neck areas [3]. In older children and adults the bites are most commonly on the limbs. Dog bites are frequently complicated with a crush injury as a result of the high masticatory forces that can be delivered by certain breeds; 310 – 31790 KPa depending on the breed [4,5]. Mandibular fractures in infants are rare. In reported jaw fractures in children under 10 years old 0.9–2.6% occur in the age group 0–1 year [6-8]. They are almost unheard of in neonates [4]. They are between 1.6 – 6 times more common in male children. In children, fractures are most common in the condylar region (36%), followed by the canine region (23%) [9]. The reported causes of fractured mandibles in infants and neonates have been due to road traffic accidents, non accidental injuries, traumatic delivery, bicycle accidents and falls.

Our literature search using mandible, fracture, child, infant, dog bite and animal bite found only 2 cases of fractures of the mandible due to dog bites in the English language publications available through med line and Google search engines [10,11]. One case report was regarding a four year old and the other a 1 year old. Fractures of other skull bones due to dog bites have been reported in children as young as 4 months old [12]. We present what we believe to be the youngest patient presenting with a fractured mandible due to a dog bite.

## Case Presentation

On the 24th April 2006 a 9 month old girl was brought to the Emergency Department of a hospital which routinely refers patients to us. She had sustained a dog bite to her face 30 minutes previously. She was seen by the emergency physicians and was found to have received multiple lacerations to her face over the right zygoma, right paranasal area, right cheek, left eye lid, left paranasal area, left lower cheek and over her left body of her mandible. She was fully examined and found to have no wounds elsewhere however due to the circumstances of patient and parental distress, intra-oral examination was not possible. The initial work up did not include radiographic investigations as it was felt by the emergency physicians that only a soft tissue injury was sustained. On the advice of the on-call maxillofacial team she had her facial wounds cleaned with aqueous iodine solution and she was started on an oral course of Co-Amoxiclav and paracetamol. An examination by the ophthalmologist revealed no ocular injury.

**Figure 1:**
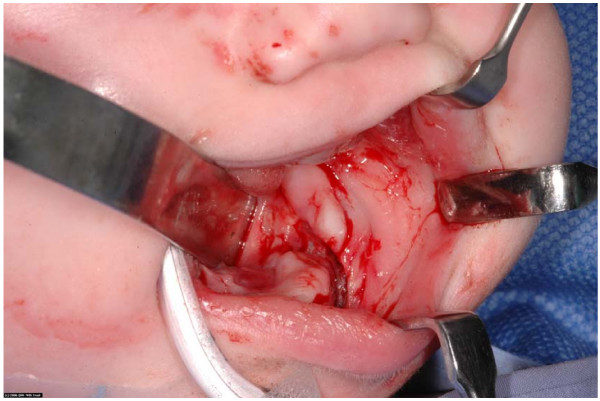
**Intraoperative view of fracture mandible**.

**Figure 2:**
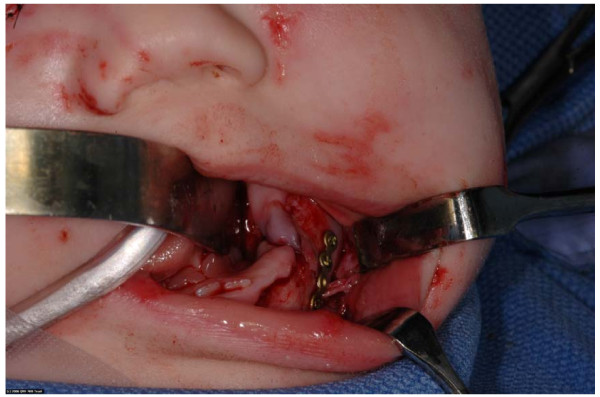
**Intraoperative view of mandibular mini-plate in situ on mandible**.

**Figure 3:**
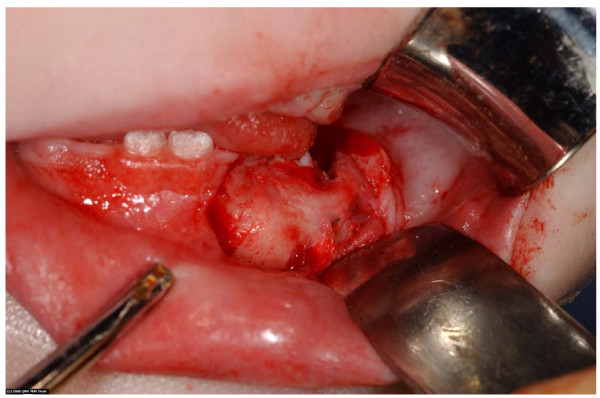
**Intraoperative view of healed mandibular fracture**.

She was transferred to our unit the following day, fasted in preparation for an examination under general anaesthesia and primary closure of her facial lacerations. During the procedure it was found that she had sustained an open fracture of her left mandible consistent with a dog bite. (fig 1). This was treated via a trans-oral approach with a five hole 1.2 mm titanium plate and four 3 mm screws. (fig 2). She had her facial laceration copiously irrigated with normal saline and chlorhexidine and primarily closed in with a fine nylon suture. She was discharged home the next day. Seven days later she attended for removal of sutures under a general anaesthetic and examination of her jaw. The facial wounds were healing well and there was no movement at the fracture site. Ten weeks after the first operation she was admitted for removal of her mandibular plate under general anaesthetic. There was good bony union and the plate was removed with no complications (fig 3). She will continue to be reviewed in clinic to monitor dentoalveolar development and mandibular growth.

## Conclusion

Dog bites account 250,000 minor injury and emergency unit attendances in the UK each year and 740 people per 100,000 are bitten by dogs 18. However only a minority seek medical attention. Overall 2.6/100,000 require hospital admission. The annual incidence of dog bites in children aged under 15 is 22/1000 [13]. In the United States of America, the annual mortality is 7.1/100 million population, with 57% occurring in children under the age of 10 years. In children, however 76% of bites are to the lips, nose or cheeks [14].

Dog bites causing mandibular fractures are extremely rare. There are only two cases previously reported. In one case a 4 year old boy sustained a right mandibular body fracture that was treated with elastic intermaxillary fixation [11]. In the other case a 1 year old girl sustained a fracture at the left angle and right body resulting in an avulsed free segment of bone that required extensive reconstructive surgery [10].

This case is interesting for several reasons; firstly dog bites in this age group are rare, secondly fractures of the mandible due to dog bites are extremely unusual and thirdly this seems to be the youngest in the literature [8,9,11,14,15].

There are a number of options with regards to treatment of mandibular fractures in children and infants [16]. The surgical principles differ from that in adults due to unerupted or incompletely formed teeth, and the relatively soft, growing mandible as well as the poorly differentiated cortical bone. However, any slight discrepancy in alignment of the mandibular bone will be compensated for in future growth. There is a risk of damaging the unerupted dentition and disturbing facial skeletal growth. We opted for open reduction and internal fixation with a titanium mini plate and screws, over the lower boarder of the mandible, with subsequent removal of these after 10 weeks. We considered the use of resorbable fixation, however the smallest resorbable mini plate would have been too large for the small size of the mandible. The fracture was mobile and displaced thus needing rigid fixation. IMF was contraindicated due to absence of erupted dentition in the region of the fracture.

The child is still under regular clinic review and is being followed up with regards to scar maturation and dental and facial growth.

This case highlights the importance of intra oral examination in facial trauma victims for clinical signs of facial fractures.

## Consent

We confirm that informed written consent was obtained from the patients parents to publish the case presentation and the non identifiable intra oral photos of the patient.

## Competing interests

The authors declare that they have no competing interests.

## Authors' contributions

TWMW – Literature Search, Wrote Report, PCM – Literature Search, Wrote Report, LC – First Edit, Wrote Conclusion, JCC – Final Edit and General Advice.
